# Reaching the ideal cardiovascular health: is this the key to preventing multiple long-term conditions?

**DOI:** 10.1016/j.lanepe.2024.100968

**Published:** 2024-06-24

**Authors:** Yogini V. Chudasama, Kamlesh Khunti

**Affiliations:** Leicester Real World Evidence Unit, Diabetes Research Centre, Leicester General Hospital, University of Leicester, Leicester, UK

Multiple long-term conditions (MLTCs) or multimorbidity, has become a major health problem globally due to an ageing population.^1^^,^^2^ However, the increased prevalence of chronic conditions is not only due to an ageing population but also due to a combination of rising prevalence of obesity and a shift to unhealthy lifestyle behaviours in recent decades.^3^ Therefore, targeting patient lifestyle behaviours could significantly help to prevent and reduce the current epidemic of MLTCs. The American Heart Association launched the concept of the ideal ‘cardiovascular health’ measured using Life's Simple 7 (LS7) and Life's Essential 8 (LE8), Fig. 1.^4^ Previous studies have demonstrated the associations between higher cardiovascular health and lower risk of single chronic conditions, and MLTCs in two studies.^5^^,^^6^ Yet, to date, there has not been a study exploring the association of the change in cardiovascular health and the risk of developing MLTCs.

**Fig. 1:**
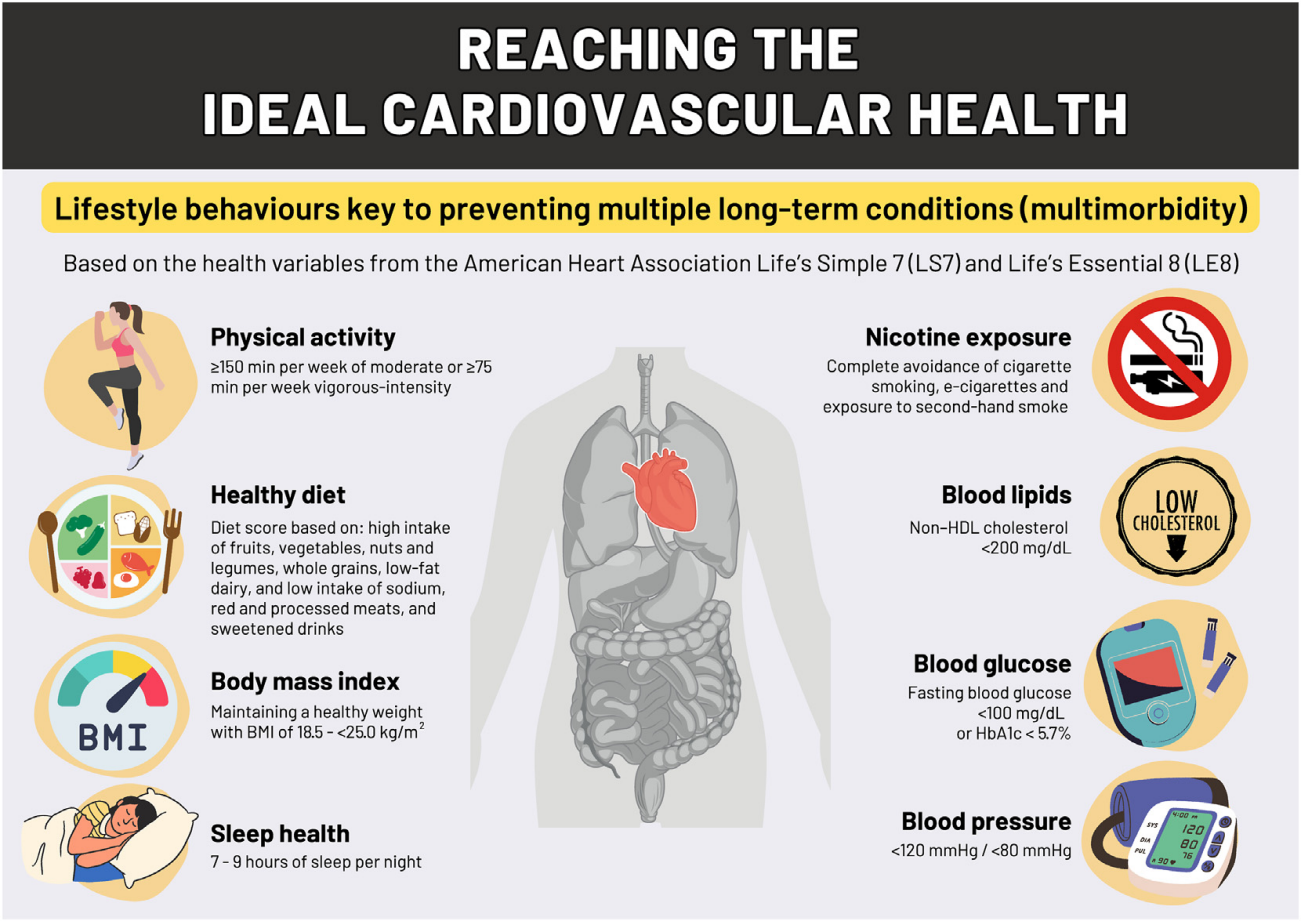
Summary of the ideal cardiovascular health based on the American Heart Association Life's Simple 7 (LS7) and Life's Essential 8 (LE8). Multiple long-term conditions (multimorbidity), two or more long-term conditions; min, minutes; BMI, body mass index; non-HDL, non-high-density lipoprotein cholesterol; e-cigarettes, electronic cigarettes.

Figure 1 was created by YC using Canva. All graphical elements used in the figure are either original or sourced from Canva's licensed library.

In this issue of *the Lancet Regional Health–Europe*, Prugger and colleagues add an important study to the literature by investigating the relationship between changes in cardiovascular health over time and subsequent MLTC risk using community-based cohorts from the UK and Finland.^7^ MLTC in the study was defined as 2 or more of 12 chronic conditions. The primary analysis was based on 9715 participants from the Whitehall II multi-wave prospective cohort study in the UK, and the validation cohort of 75,377 participants from the Finnish Public Sector cohort study in Finland. The main finding was that both LS7 and LS8 assessments were associated with a lower risk of incident MLTC. In the UK cohort, the risk of MLTC decreased by 8% (hazard ratio 0·92, 95% confidence interval 0·88–0·96) per one ideal LS7 metric increase over 5 years and by 14% (0·86, 0·80–0·93) per 10-point increase in LE8 score over 10 years. These results were robust to comprehensive sensitivity analyses and confirmed in terms of change in cardiovascular health over 4 years in the validation analysis. For both Whitehall II and Finnish study populations, the population preventable fractions (PPF) indicated that achieving ≥2 ideal LS7 metrics based on the corresponding hazard ratio and prevalence was estimated to prevent 53% of individuals from developing MLTC who initially lacked any LS7 metrics.

The main strengths of this paper were specifically the longer length of follow-up (median 31·4 years), observing incidence of MLTCs, using both LS7 and LS8 assessments, validation cohort, and several sensitivity analyses. However, this study is not without limitations; the diet component was missing from the validation Finnish cohort, and no other proxy was used to replace the missing diet variable. Additionally, the Whitehall II cohort was over-represented by people of White ethnicity, men and British civil servants working in London offices, thus the findings limit generalisability to other social and ethnic groups. There has been greater attention on ethnic and social health inequalities due to the higher prevalence of MLTCs in these populations,^8^ thus further research on cardiovascular health and the risk of MLTC may benefit from larger national datasets with stratified analyses to identify whether the findings differ between patient demographics. The assessment scores could therefore be further developed and tailored to suit these populations as they have different dietary patterns than those in the US, vary in physical activity levels, and the ethnic minority populations differ in body mass index compared to White Caucasians. Lastly, the study focused on the development of 12 chronic conditions over time. We agree with the authors that a larger list of conditions makes the concept meaningless for clinicians and for identifying prevention targets, though, the current literature holds inconsistent definitions and approaches to measuring MLTC.^9^ For this reason, future studies relating to MTLC would benefit from using the definition of MLTC defined by the Delphi consensus study from a panel of international experts that selected 24 main conditions, to help facilitate consistency and comparison of studies.^10^ This may also help prevent non-cardiovascular related MLTCs.

MLTCs affect each individual at some point in their life, either personally or through family, friends, or work. It has a major impact on quality of life as it deteriorates over time, leads to poorer health outcomes, and accounts for a higher healthcare workload, costs, and mortality. This new study provides us with evidence indicating that reaching the ideal cardiovascular health could potentially have prevented over half of the participants from developing MLTC. Therefore, we should start focusing on the immediate causes which are obstructing individuals from reaching their ideal cardiovascular health to be able to minimise and prevent future diagnoses of MTLCs.

## Contributors

YC–Writing original draft & figure design.

KK–Writing, review & editing.

## Declaration of interests

KK is the National Lead for multiple long-term conditions (MLTC) for the National Institute for Health Research Applied Research Collaboration (NIHR ARC), Co-Director of the NIHR Global Centre for MLTC and Co-Chair of the National MLTC Cross NIHR Collaboration.

YC has no competing interests.
